# Exsection of the Right Superior Maxilla, and a Portion of the Left, for Disease of Long Standing

**Published:** 1860-07

**Authors:** Wm. H. Gott

**Affiliations:** Readstown, Wisconsin.


### ARTICLE XI.

*Exsection of the Right Superior Maxilla, and a portion of the Left, for Disease of Long Standing.* By Wm. H. Gott, M. D., of Readstown, Wisconsin. (Communicated by Jas. M'Naughton, M. D., of Albany, N. Y.)

I was consulted in April last by Peter Guist, aged twenty-three years, respecting a tumor of the right superior maxilla. From his own account, it appears that while residing in Morgan County, Ohio, ten years ago, a tumor of the size of a small pea was observed to be growing on the under surface of the jaw, behind the canine teeth, and attached to it by a delicate pedicle. After the lapse of a year or so, when the tumor had attained to the size of a small marble, a physician was consulted, who advised its removal without delay; it was accordingly snipped off by the scissors close to its point of attachment to the bone.

For three or four months nothing more was seen of it, and the hope was indulged that its removal would prove effectual; it, however, shortly reappeared at the site of the attachment of the pedicle to the bone, and increased in size gradually, so that by the end of a year, the alveolar process as far as the first molar tooth had become implicated.

In the fall of '51 or '52, he emigrated to Badax county, in this State. In '54 or '55, he submitted to an operation at the request of a physician, under the promise of a speedy and permanent cure. The operation had recourse to, as far as I have been able to learn, consisted in the extraction of a few of the teeth and the shaving off of the body of the tumor from the bone.

The hemorrhage following this operation was very profuse and was with great difficulty controlled; no benefit was experienced, for the tumor soon began to enlarge with more rapidity than formerly.

The foregoing history is imperfect in its details, but is as full as could be obtained from the patient. He first came under my observation in the early part of the summer of '57, soon after I commenced practice in Readstown, and his condition both general and local, was noted as follows:

A tumor, commencing just below the orbit, with a slightly elevated and abrupt edge, extending downwards and projecting out of the right side of the mouth, which is drawn to the same side, distends the right cheek to a great degree, giving rise to deformity, and also much inconvenience to the patient; the entire palate portion of the maxillary and palate bones is involved in the morbid growth, which projects from the hard palate to such a degree as to force away the lower from the upper maxilla, and thus to render it difficult for the patient to masticate his food properly.

The tumor is distinctly limited to the jaw, and does not extend beyond the limits of the bone in which it has evidently originated; is painless to the touch; firm and unyielding, except at the conical shaped point where it projects from the mouth; a sensation of deep seated elasticity is here imparted to the finger, as if the tumor was covered by a thin shell of bone which yielded when pressed upon, and appeared to recover itself upon the removal of the pressure.

Its surface is nodulated, and covered with a thick white membrane, over which numerous small blood vessels ramify, with one or two spots of superficial ulceration which furnish a little pus; pressure on the hard palate of the left side and on the tumor on the right side detected no yielding of the bone; in fact, in the latter situation, the sensation of bony hardness was imparted to the finger; the teeth, three or four of which remain, have become irregular and projecting, but are still firm in their sockets.

The growth of the tumor thus far has been slow, unattended either by pain, hemorrhage, or any appreciable derangement of the general health.

The patient was advised to submit himself without delay to the operation of excision of the greater portion of the maxillary bone, as the only course which would afford him any chance of relief. He, however, objected to undergo such an operation, and for no very satisfactory reason, and consequently passed temporarily from under my observation.

In April last he reapplied to me, fully satisfied his case would soon be beyond surgical relief if let alone, and willing to undergo any operation which would afford him a chance of his life. A careful and minute examination of his case was had, when it was found that the chances of a successful result were not as favorable as at the time of his first application. The tumor had made considerable progress, and was now found to embrace the following parts: Commencing at the inner angle of the eye, its abrupt and well defined edge, which at the first examination two years previous, was felt half an inch below the margin of the orbit, was now perceived to be on a level with it, and to reach to the junction of the maxillary with the malar bone, also to have encroached upon the side of the nose; the alveolar ridge of the left maxilla as far as the first molar tooth was found to have become implicated in the disease, as well as a portion of the hard palate of the same side to about one-third of its extent.

The tumor in its general appearance had not changed materially, excepting in its increase in size, and in the fact of its surface having become somewhat more nodulated with a greater number of vessels of larger size ramifying over it.

With the view of ascertaining, as far as possible, the nature of this growth before resorting to any operation, the exploring needle was introduced into its most prominent part, where the sensation of indistinct fluctuation was first perceived, and it appeared to enter a softish mass without resistance; it was then found that a considerable degree of *lateral motion* could be given to the instrument: a small quantity of blood followed the withdrawal of the instrument; the remaining portions of the tumor were explored in the same way, but into which the needle entered not without force, and into a dense substance where no *lateral motion* could be obtained, except with such a degree of force as would have been unjustifiable to employ.

For the past few months his general health has been on the decline, and is still so, owing partly, as he thinks, to the imperfect mastication of his food, and partly to its quality, it having been of a liquid nature mostly for some time; he is now somewhat emaciated, and incapable of the physical exertion necessary to earn a livelihood.

I was well aware of the importance of a correct diagnosis respecting the simple or local and non-malignant nature of this growth, also of the inadvisability of resorting to an operation should this be judged a case of medullary sarcoma. The diagnosis as to the growth being of the first variety was based upon the following facts:

1st. There has been but little constitutional derangement attending the growth and development of this tumor from the first; that which has of late manifested itself is undoubtedly due to causes independent of malignancy.

2d. This tumor is distinctly limited to the maxillæ, with well defined boundaries, and unlike a malignant one, which for the most part incorporates itself imperceptibly with surrounding parts. Its slow uniform growth, its firm unyielding nature, (except at one point,) the absence of pain at all times and hemorrhage, its lobulated surface, and the fact that no fungous growth has sprouted from the superficial ulcerations, nor any fetid discharge escaped from them; these circumstances, independent of the light derived from the employment of the exploring needle, combined together, render it probable that the disease is local, (if not entirely so, and if from a simple, it has insensibly merged into a malignant form in part, such a change is as yet in its early stage,) and that the performance of an operation without delay would be in accordance with good surgery, and followed by a permanent cure.

The simple nature of this tumor having been determined upon, I advised the patient to be operated upon at an early day, and the 4th of May, 1859, was fixed upon. After a few days of preparatory treatment the operation was performed as by appointment, as follows: After partial anæsthesia had been induced by a mixture of equal parts of chloroform and ether (it was not deemed prudent to put the patient under the full effect of the anæsthetic, from fear that death from strangulation from the blood passing into the windpipe might take place,) an incision was made from the external angular process of the frontal bone to the corner of the mouth. A second was then carried from the nasal process of the superior maxillary bone to the mouth near the mesial line. The tumor was now laid bare, by dissecting upwards the tissues of the cheek to the orbit into which the dissection was pursued, and its tissues detached from the floor.

The nose was next separated from the bone and held over to the left side by an assistant. This dissection completed, the operation was suspended for a few minutes, in order to secure the arteries, and to allow the patient to recover from the effect of the anæsthetic.

The tumor was now fully exposed to view. The operation having been resumed, the malar bone was divided near its middle by the metacarpal saw—then the nasal process of the maxillary by the bone cutters and chisel; the saw introduced into the nostril, the alveolar ridge was sawn through, and the hard palate to the attachment of the velum palati. The tumor being now forcibly moved and loosened by the finger introduced from behind, was lifted out after a little delay in dividing its remaining attachments. The large cavity thus exposed was immediately filled with small sponges to arrest the profuse hemorrhage, which done, the implicated portion of the left maxilla was separated by the saw and bone cutters.

The patient, much exhausted from loss of blood together from the shock of the operation, was allowed a little brandy with thirty drops of laudanum, which, with the application of dry warmth, brought about gentle reaction in a little while. The wound was allowed to remain open exposed to the air for nearly an hour, the more to guard against secondary hemorrhage, when the cavity was filled with balls of lint and the flap stitched up, and the patient removed faom the table to his bed. The only dressing applied consisted of pledgets of lint wet in cold water.

Three or four hours after the operation several small clots of blood were thrown off from the stomach, when an additional dose of opium was given to allay restlessness and pain. In forty-eight hours after the operation the sutures were removed and union by the first intention had taken place throughout the incisions. Under the free use of animal broths the patient gained in strength so rapidly as to be able to sit up out of bed on the eighth day, and at the end of another week to be about on his feet.

The general condition and health of the patient six weeks from the time the operation was performed, when he passed from under my care, appeared to be good, and were improving from day to day. The partial paralysis of the cheek consequent upon the division of the branches of the facial nerve gradually wore away, and its sensibility became fully restored. At the time of writing this report, nearly four months after the operation, the general health of the patient is better than it had been for the past year or two, and he is now engaged in his vocation, that of a farmer. His articulation is very distinct, and the process of mastication can be performed without difficulty.

I will now describe the appearance of the tumor as accurately as possible. Its weight three or four hours after the operation, was seven ounces and a half. Its greater portion was composed of a dense, more or less fibrous substance of a whitish and yellowish white color. Its central portion, where the indistinct fluctuation was perceived before removal presented a very different character, and was made up of a structure of a soft medullary nature, and very vascular. Beside the thick membranous capsule investing the tumor, and beneath it, a thin lamina of bone was spread out over its circumference, constituting the walls of the antrum.

*Note.*—In a letter to Dr. M'Naughton, dated August 29th, 1859, Dr. Gott adds the following postscript:

"I have seen my patient several times lately; he says his health is better at present than it has been for a year or two past. He is now earning his own livelihood at his occupation, that of a farmer. The parts have entirely healed, and with the exception of some falling in of that side of the face and a slight drawing down of the lower eyelid, no evidence that such a tumor had been removed from the face would exist."—*Amer. Jour. Med. Sci.*

---

